# Notes and Comments

**Published:** 1923-04

**Authors:** 


### NOTES AND COMMENTS.

### THE TWO MINISTERS.

SIR ARTHUR GRIFFITH-BOSCAWEN has had a brief and hapless career as Minister of Health, and his defeat at Mitcham has compelled his resignation before he had the smallest opportunity of showing his mettle in Whitehall. General sympathy has been extended to a politician who has done well in other offices but whose electoral experiences have been singularly unhappy—in less than two years he has fought four elections and lost three. His successor, Mr. Neville Chamberlain, who moves from the Post Office to Whitehall, has had an experience of municipal administration in Birmingham which should be of the utmost value to him at the Ministry of Health. He has not only been Birmingham's Lord Mayor, but Chairman of its Town Planning Committee, and with Town Planning his relations will now be intimate and peculiar. He has already publicly declared that public health is the one subject, above all others, in which he is interested. He recognises that improvement must necessarily be slow, but knowledge, experience and determination can work wonders. Should he be granted reasonable length of office, Mr. Chamberlain will enjoy opportunities which have been denied to his predecessors. No Minister dealing with home affairs has a greater or more important task, especially in relation to the burning subject of housing, which had so *funeste* an influence upon the electoral fortunes of Sir Arthur Griffith-Boscawen.

### YOUTH IN THE PUBLIC-HOUSE.

IT is impossible to escape the significance of the large majority by which the House of Commons has given a second reading to Lady Astor's Bill prohibiting the sale of intoxicating liquor to persons under eighteen. Already spirits may not be sold to young people under sixteen, and mere children are excluded from public-houses altogether, but any boy or girl of fourteen may walk into a licensed house and, like David Copperfield, demand a pint of stout "with a good head to it." With the nicest regard for liberty, it is difficult to find an argument for the continuance of such a state of things as that. Alcohol, even the minute proportion of it which manages to make its way into modern beer, is not good for people in their teens, nor is the atmosphere of the average public-house any better for their *moral* than the contents of its barrels. It is perfectly true that the real solution of the drinking problem is the creation of a new type of public-house to which any man, in any class, might take his wife and children; but pending that consummation Lady Astor's Bill is a help to the teaching of commonsense in this matter. In the ordinary way a private member's measure of this kind would enjoy little chance of becoming law, but since it is clear that very much more than half of the members of the House of Commons are anxious to see it passed, the Government may think it wise to provide facilities for its further stages, notwithstanding the Home Secretary's doubts of the possibility of enforcing it. But those doubts are surely exaggerated.

### FOREIGN HEALTH OFFICERS.

IT is impossible not to feel some compassion for the large company of foreign health officers who are paying a visit of inquiry to this country under the auspices of the Health Organisation of the League of Nations. Their visit has been extremely interesting for them, but it has also been extremely strenuous. In London they have been initiated into the administrative methods of the Ministry of Health, and the statistical system of the Registrar-General's department. They have learned all the secrets of the Metropolitan Asylums Board and the Water Board, and have heard much about the housing activities of the County Council. They have, however, been able to do their work in the provinces under somewhat less breathless conditions. They have divided themselves into sections which have paid, and, indeed, are still paying, prolonged visits to Birmingham, Glasgow, Manchester, Liverpool, Bradford, and Newcastle-on-Tyne. At the end of their six weeks' tour the delegates, who represent seventeen countries, will have a conference at Geneva, which is becoming as famous for conferences as it long has been for watches and Calvinism. Their collective impressions will be highly interesting—we know that one of them has already likened Glasgow to Heaven. This is a new light upon Glasgow, and probably upon Heaven, and the least the Second City can do is to present this distinguished explorer with its freedom. There can, however, be no doubt that a visit of this kind, so well planned by the Society of Medical Officers of Health, and so thoroughly carried out, will be of the utmost advantage to the delegates themselves and the countries in which they work. The expenses are being paid by a contribution of £12,000 a year for three years made by the Rockefeller Foundation to the League of Nations. If the League has not yet been able to secure the peace of the world it is assuredly doing something to improve its health.

### THE DANGEROUS DRUGS BILL.

MOST Parliamentary measures are capable of improvement, both before and after passing, and the Dangerous Drugs and Poisons (Amendment) Bill, which has passed its second reading in the House of Commons, is clearly no exception. Its main provisions are, however, admirable, and we sincerely hope that it will become law during the session—happily it is a Government measure. It has been complained that the Bill is drastic, but that is the best of reasons for supporting it. Legislation dealing with the highly remunerative traffic in noxious drugs sorely needs to be drastic, and a fine of £1,000 or ten years' penal servitude, or both, is not a whit too severe a penalty for traffickers who work untold physical and moral injury. But the Bill does more than increase penalties. It greatly augments the existing powers of the police, enables the premises of suspected persons to be searched, and brings within its purview acts done in this country with a view to illicit transactions abroad. There is, however, a blot upon it. One of its clauses appears to make it difficult for a medical man, in certain circumstances, to obtain scheduled drugs, and Sir S. Russell-Wells obtained from the Home Secretary an undertaking so to amend its language as to remove all ambiguity. It would be intolerable that a qualified practitioner should be unable to obtain any drug at any time. For the rest there is no fault to find with a measure which cuts at the root of the detestable traffic about which we wrote last month.

### THE IMPROVING DEATH-RATE.

IN the leading article in our March number on "The Conquest of Disease," we commented upon the gratifying fact that in 1920 and 1921 the infantile death-rates were the lowest on record. The Registrar-General's figures for last year, which have since been published, show a death-rate of 77 per thousand, which leaves the 80 of 1921 well behind, and constitutes a fresh "record." This is still too high, but the improvement has been rapid, and we may hope that it will now also be continuous. The rate was very much kept down by the absence of infantile diarrhœa, but that immunity was considerably neutralised by outbreaks of measles, influenza and bronchitis. On the other hand, the general death-rate was a fraction higher than in 1921, which itself was a fraction worse than 1920. The improvement upon the year immediately preceding the war is, nevertheless, substantial, and clearly emphasises the undoubted fact that the national health grows more robust. Simultaneously with this amelioration there is a further fall in the birth-rate. What is lost at the beginning is gained at the end, which may, or may not, be consolatory, according to the point of view. The condition of the birth-rate undoubtedly gives cause for anxiety on general grounds, though the density of our population, *vis-à-vis* its means of support, has already reached a point which justifies apprehensions of another kind.

### THE LEGITIMACY BILL.

MR. H. B. BETTERTON'S very modest Bill for legitimising children born out of wedlock whose parents marry either before or after it becomes law, has passed its second reading with the blessing of the Government and without a division. It may, as the Attorney-General suggested, need some amendment in Committee, but the very simplicity of its aims should make its passage comparatively easy. We had something to say last month of the unhappy position of the illegitimate child, who is at present compelled to go through life with either a stigma or a secret. That stigma the Bill seeks to remove, while, however, postponing children born before their parents' marriage to those born after it. It was thus sought to avoid difficuties about the succession to property and titles, to say nothing of appeasing the wrath of Sir Frederick Banbury. Side by side with this measure Captain Bowyer has introduced a Bastardy Bill to increase the amount payable under an affiliation order and to appoint collecting officers. This suggests elaborate and costly machinery, and the Bill is less likely to obtain favour than Mr. Betterton's attempt to secure justice for tens of thousands of children whose chances of health and a useful life are seriously jeopardised by a heartless law which legally marks them as nobody's children, and places the entire onus of their upbringing upon a mother who is usually little able, and is often very unwilling, to do her duty by them.

### THE CANCER RESEARCH OF TO-DAY.

IN view of the despairing call for a "cure for cancer," it is a pity that the "Lancet's" recent review of the present position could not have been made available to the public also. It is, of course, well known that several workers have succeeded in producing cancerous growths in the skin of animals by repeated application of some moderately vigorous irritant, such as tar or its extracts. This may seem a very indirect step on the road towards "cure." Mere experimental product of the growths is, however, only half the story. The rest is vital towards developing a scheme of practical prevention and, as such, should also be known. In the first place, it is found that the first sign of the tumours may not appear until long after the irritant has ceased to act. The cells once irritated may have developed a tendency to produce growth long before they actually do so. There is, in fact, a "latent period," and the existence of these two factors—long continued irritation and often a long latent period as well—probably accounts for the fact that growths usually do not occur until the second half of life. Also the connection of previous injury to ultimate growth development is explained when it is found that previous damage to the skin, by itself quite incapable of producing a tumour, greatly accelerates this formation when a suitable irritant is afterwards applied. Another extremely important point is that in mice which have once developed a tar cancer and been cured by operation, a second cancer can never be produced however long further tar irritant be applied. Apparently they become immune. The deduction probably is that, if the first growth be completely removed, we can have cancer but once. This may be poor consolation, but at least it shows one form of immunity to the disease and, therefore, may well be the first step towards the discovery of many others. An artificial immunity to cancer—as to smallpox—would be the greatest boon medicine could ever confer.

### LONDON'S HEALTHIEST WINTER.

AS we near its end the fact becomes more certain and, indeed, more remarkable, that this is the healthiest winter London has ever known. To whatever disease we turn, the same relative scarcity is seen. From November onwards is usually the bronchitis and pneumonia season, but this year there was hardly any sharp rise in the number of cases noted. The death-rate at the moment stands at a figure usually associated with early summer. The fever hospitals have rarely had such unique opportunity of real spring-cleaning. Relatively speaking, there is no measles and no scarlet fever, and diphtheria has been remarkably scarce. In contrast with last year, when these diseases positively raged, the present picture is most striking. The cause of this happy state of things is difficult to assign. It is true that outbreaks of infectious disease run in definite cycles of time; they recur at more or less regular intervals. But it is unusual for all of them to disappear together. One explanation may be that influenza is the real keystone of such general outbursts as the past has seen. Even in the absence of a great influenza epidemic the disease may be sufficiently subtly prevalent to pave the way for the others, which, once started, flare up according to their own particular habit. In any case, he would be a brave man who would say that this healthy winter is a forerunner of many more to come. Whatever means of prevention already exist must in nó degree be relaxed, for the probability is that this remarkable public immunity from all health troubles is a coincidence and must be treated as such.

### EFFECTS OF REMOVING TONSILS.

IN spite of the heated arguments which have raged round this subject, the fact remains that surgical removal of tonsils is still a very frequent procedure. Whether indiscriminate removal in children is altogether justified will soon be made clear by publication of statistical details of the after-effects from some of the great hospitals. The point is one which parents will be glad to see settled. Meanwhile we note in the professional journals an authentic list of end results in the case of 130 adults who had undergone the operation to free themselves from attacks of tonsillitis and its effects. The method of operation was exactly the same in each case, and the whole of the tonsillar tissue had been carefully removed. Reviewing the figures there is no shadow of doubt that all those patients whose previous tonsillitis had resulted in rheumatic symptoms were greatly relieved and mostly cured entirely. When the complaint had been simply the septic state of the mouth and pharynx, equally great relief was obtained. The proportion of success is undeniably great. One curious point emerges, however. A few of the patients complained that their singing voice had been decidedly weakened, although their health itself was far better.

### INDUSTRIALISM AND PHTHISIS.

THE relation of tuberculosis to industrialism is so intimate and complex that practically the whole of the March number of *Tubercle* has been devoted to this subject. There are more questions than answers in this number. Why, for example, should the tailoring, shoemaking and printing industries be so phthisis-ridden? Apart from their heavy phthisis morbidity, these industries are not exceptionally unhealthy; indeed, their position in other respects is comparatively favourable. An answer—not necessarily the right one—is that there is more overcrowding in these three industries than in most others. In other words, there is a greater chance of one factory hand infecting another than in other industries. Opposed to this explanation is the Andvord-Römer hypothesis (it might almost be called a law) that the phthisis of adult life is the terminal stage of a disease contracted in childhood, the fate of the patient having been decided decades earlier by a massive infection in childhood. Trades are largely hereditary, and the tailor, printer or shoemaker who develops phthisis may have caught it in his childhood from his father. But why did the father contract phthisis in his turn? If we find that these three industries predispose to tuberculosis simply because of overcrowding and consequent increased facilities for infection of one adult by another, the remedy lies to hand. Legislation must provide better accommodation and stop overcrowding. But if the main factor is to be sought elsewhere the best course to pursue would probably be to remove from these industries workers found to be suffering from tuberculosis in an early stage—in any stage for that matter. Pulmonary tuberculosis is so remarkably amenable to treatment in the early stages that early diagnosis is more than half the battle. It has recently been shown that at the Mt. McGregor Sanatorium in the U.S.A., 90 per cent. of its patients admitted in the incipient stage of the disease were found to be still fit for work from one to seven years after discharge.

### VITAMINES VERSUS MINERAL SALTS.

SINCE the magic word "vitamine" first came into public use there have been many fluctuations in the accepted value of these substances in nutritional economy. So large a number of experiments has been carried out on domestic animals that we are apt to accept the results obtained with them as unreservedly applicable to men and women also. Nevertheless, the first Report of the Rowett Institute in Aberdeen gives cause for reflection. Among themselves animals, according to their species, are found to show remarkably different effects when deprived of vitamines. Some, particularly farm animals, have such low vitamine requirements that, when receiving any ordinary diet, they could practically never themselves suffer from their lack. In fact, it might be quite possible for a cow to continue in perfect health when itself taking so little vitamine that its milk was practically vitamine free. The result, for a child fed on this milk, need not be explained. In some beasts, such as pigs, mineral salts are far more important than vitamines. Deprived of the former they show obvious malnutrition in a month, while loss of the latter affects them amazingly little. Rickets was produced in pigs by taking away their supply of salts, but, however little vitamine they received, no such result was seen. Rickets in animals as well as children would seem to depend upon all three of the factors, low vitamine diet, deficiency of mineral salts and lack of sunlight, and not upon any special one of them. In a particular species, one or other factor may be of special importance, but the predominant factor is not the same for all.

### PORRIDGE AND INDIGESTION.

DR. JOHNSON, in one of those moods of sardonic humour which can only be reached by the truly great, defined oatmeal as a food given to horses in England and to men in Scotland. But Dr. Harry Campbell—his name bewrayeth him—has been telling the Institute of Hygiene that the teeth of the Scot, despite the food of which he ruthlessly robs the hungry horse, are even worse than those of the Englishman, which are notoriously bad. He would, therefore, abolish porridge, and for that those who dislike it or who are disliked by it, will thank him. But he would ban puddings also, which is more serious. He is plainly right, however, when he says that soft foods are the enemies of strong teeth and robust digestions. "Well-baked, crusted bread," he adds, "is admirable for the digestion"—a statement which will please the correspondent who writes to us this month about the bad character of English bread. Our bread is not well-baked, and is, consequently, not crusty, and we love to have it so. Pasty bread and plenty of sugar are the idols of vast numbers of exceedingly foolish persons who would be the better for gnawing a bone. Then they would probably have teeth as good as those of the average dog. The war-time regulation which forbade the selling of new bread was a blessing which we shed in haste, and the dentist has been busier than ever during the last four years.

### DIRTY MONEY.

MONEY has often been called "dirty," and in America they are finding it to be actually "filthy." In that enlightened land the bank which sends worn and soiled paper money to the Treasury to be exchanged for crisp new notes, has to bear the cost of remitting it to Washington as "unfit currency." We should have supposed that the cost would be no greater than was represented by a few postage stamps, but, whether the expense be great or small, everyone is shy of incurring it. The result is that a vast number of dirty notes are in circulation. Moreover, the dirtier they are the more rapidly they circulate, each holder being anxious to get rid of them with the utmost expedition. Nor are things any better when the paper reaches the banks. They start circulating it among themselves, so that some other bank may bear the cost of converting the old into the new. The dangers of constantly handling germ-infected paper money are obvious, and now that New York's Health Commissioner has become a member of the Senate he will perhaps see the wisdom of adopting the English system of exchanging old notes for new without cost to anybody. Bank of England and Treasury notes are the cleanest paper money in the world. It would seem, indeed, that the average circulation of a Treasury note is barely a month, save perhaps in remote parts of the country where coins also circulate for a longer period than in great agglomerations of population.

### HEIGHT AND WIDTH.

IN Aldwych, the fine London thoroughfare which in its name commemorates the old Wych Street, there has just been erected a great block of offices, 124 feet high, which it is expected some day to increase to 144 feet by the erection of a central tower. There has of late been an agitation for a considerable increase in the height of buildings in the heart of the capital, and in this case the London County Council has used the discretionary powers in this respect with which it has been invested by Statute. Hitherto, we believe, the limit had been 80 feet. Both health and amenity may be injuriously affected by unduly high buildings, and any attempt to acclimatise the New York type of "skyscraper" would have to be resisted strenuously. From the point of view of health everything depends upon the width of the street, and there are not many streets either in London or the great provincial towns wide enough to endure really lofty buildings. We have nothing to compare, for instance, with the Champs Elysées, and even there the buildings are not especially tall. The result of high buildings in a comparatively narrow street may be seen in such a thoroughfare as Northumberland Avenue, which is little better than a windy tunnel. There are good, bad and indifferent winds, just as there are good, bad and indifferent streets, and when a bad wind rages in a bad, tunnely street both health and temper are likely to suffer.

### SANITARY MILKING.

THE milking, with clean hands, of a clean cow in clean surroundings, does not seem a particularly exacting requirement. And yet, in a vast number of cases this modest minimum seems as far away as ever from attainment. There is little wonder that Dr. Robertson, the Medical Officer of Health for Birmingham, in a recent address to the Royal Sanitary Institute, emphasised the importance of educating the farmer in the elementary matter of cleanliness. There is, he said, ample evidence that without any serious alteration to his premises the farmer could produce a relatively clean milk if he adopted certain simple inexpensive methods, such as seeing that the cows' udders are clean, that the hands of the milkers are clean, that the garments worn by them are clean, and that the milk is milked into a covered or partly-covered pail. When, however, Dr. Robertson goes on to say that, while a great deal of improvement is possible in the direction of making the cowshed healthy so far as the cows are concerned, this is not essential to the production of a reasonably clean milk, we can hardly suppose that he is minimising the importance of clean, well-ventilated cowsheds. It may be that cowsheds could well be dispensed with altogether, but as they exist they must be kept clean and healthy, and this applies to the yards and the general environment. There is not only the question of the difficulty of keeping clean if the milker who may have made a clean start is in actual contact with unclean cows, but the psychological effect of unwholesome surroundings which will bring about a rapid deterioration in his habits.

